# Supplementary material for: Neonatal Outcomes after Maternal Biomarker-Guided Preterm Birth Intervention: The AVERT PRETERM Trial
Source: Diagnostics (Basel). 2024 Jul 9;14(14):1462. doi: 10.3390/diagnostics14141462 (PMC11275486; doi:10.3390/diagnostics14141462)
Supplement: Supplementary file 1 [file diagnostics-14-01462-s001.zip › diagnostics-3069770-supplementary.pdf]

Supplemental Materials

Neonatal outcomes after maternal biomarker-guided preterm birth intervention: the AVERT PRETERM trial

Matthew K. Hoffman; Carrie Kitto; Zugui Zhang; Jing Shi; Michael G. Walker; Babak Shahbaba; Kelly Ruhstaller

Contents

Table S1. Neonatal morbidity and mortality index (NMI) scoring and morbidity definitions. . 1

Table S2. Outcomes, populations, and analysis groups used in the AVERT PRETERM trial. . 2

Table S3. Results of hypothesis tests for the co-primary and co-secondary outcomes. 3

Table S4. Sensitivity analysis of neonatal morbidity and mortality index score (NMI) severity level probabilities and percent differences between arms for a range of covariate values. . 4

Table S1. Neonatal morbidity and mortality index (NMI) scoring and morbidity definitions.

**Composite Neonatal Morbidity Index (NMI) Scale***	
0-to-4 scale integrating neonatal intensive care unit (NICU) length of stay	0 = No events<br>1 = One event for (RDS, BPD, IVH Grade III or IV, any PVL, proven sepsis, or NEC) or 1-4 days in the NICU, and no neonatal mortality<br>2 = Two events or from 5-20 days in the NICU, and no neonatal mortality<br>3 = Three or more events or >20 days in the NICU, and no neonatal mortality<br>4 = Neonatal mortality
**Definitions of Neonatal Morbidity**	
**Category**	**Description**
Respiratory distress syndrome (RDS) or Hyaline membrane disease (HMD)	Requires both diagnosis and oxygen therapy<br>Must include:<br>Oxygen therapy (fraction of inspired oxygen (FiO2) ≥0.40) until infant death or ≥24 hours or continuous positive airway pressure (CPAP) and<br>Clinical diagnosis of RDS or HMD
Bronchopulmonary dysplasia (BPD)	Treatment with >21% oxygen for at least 28 days, or<br>Oxygen dependence after 36 weeks post-conceptional age
Intraventricular hemorrhage (IVH)	Determined by cranial ultrasound or computed tomography<br>Grade III intraventricular hemorrhage with ventricular dilatation<br>Grade IV intraventricular hemorrhage with ventricular dilatation and parenchymal extension
Periventricular leukomalacia (PVL)	Determined by cranial ultrasound<br>Any PVL<br>Cystic PVL
Sepsis	Must include:<br>Blood culture-proven sepsis, and<br>A clinically ill infant with infection defined as:<br>Bacterial sepsis of the newborn<br>Streptococcal sepsis<br>Severe sepsis
Necrotizing enterocolitis (NEC)	Stage I: Other – Suspect (treatment was observation)<br>Stage II: Clinical – Definite (treatment was medical)<br>Stage III: Surgical – Advanced (treatment was surgical)
Neonatal mortality	Neonatal death within 28 days of delivery

* Hassan SS, Romero R, Vidyadhari D et al. PREGNANT Trial. Vaginal progesterone reduces the rate of preterm birth in women with a sonographic short cervix: a multicenter, randomized, double-blind, placebo-controlled trial. Ultrasound Obstet Gynecol. 2011 Jul;38(1):18-31. doi: 10.1002/uog.9017. Epub 2011 Jun 15. PMID: 21472815; PMCID: PMC3482512.

Table S2. Outcomes, populations, and analysis groups used in the AVERT PRETERM trial.

Analysis	Endpoint(s)	Population	Analysis Group
**Primary**	NNLOS	mITT	Quantile
	NMI	mITT	All
**Secondary**	NICULOS	mITT	Quantile
	GAB	mITT	Quantile
**Sensitivity/Exploratory**	NNLOS	mITT, ITT	All, <32 weeks GAB
	NMI	ITT	All
	NICU LOS	mITT, ITT	All, Admitted to NICU
	GAB	mITT, ITT	<32 weeks GAB
	PTB	mITT, ITT	<37, <35, <32, <28 weeks
	sPTB	mITT, ITT	<37, <35, <32, <28 weeks

GAB, gestational age at birth; ITT, intent-to-treat population; mITT, modified intent-to-treat population; NICU, neonatal intensive care unit; NICU LOS, NICU length of stay; NMI, neonatal morbidity and mortality index score; NNLOS, neonatal hospital length of stay.

Table S3. Results of hypothesis tests for the co-primary and co-secondary outcomes.

**Co-primary endpoints**					
**Neonatal length of hospital stay*§**	**Hazard ratio**	**Lower confidence limit**	**Upper confidence limit**	***P*‡**	**Reference arm**
Historical vs prospective arm	0.73	0.58	0.92	0.01	Prospective
Maternal age	1.00	0.99	1.01	0.75	
Nulliparous vs parous	1.06	0.92	1.22	0.39	Parous
Without vs with opioid use	0.64	0.55	0.75	<0.001	With
**Neonatal morbidity and mortality index score¶**	**Odds ratio**	**Lower confidence limit**	**Upper confidence limit**	***P*‡**	
Prospective vs historical arm	0.81	0.67	0.98	0.03	Historical
Maternal age	1.01	1.00	1.02	0.13	
Nulliparous vs parous	1.72	1.53	1.93	<0.001	Parous
Without vs. with opioid use	0.38	0.29	0.51	<0.001	With
**Co-secondary endpoints**					
**NICU length of stay*§**	**Hazard ratio**	**Lower confidence limit**	**Upper confidence limit**	***P*‡**	
Historical vs prospective arm	0.83	0.66	1.05	0.12	Prospective
Maternal age	1.00	0.99	1.01	0.78	
Nulliparous vs parous	1.04	0.91	1.19	0.57	Parous
Without vs with opioid use	0.42	0.31	0.59	<0.001	With
**Gestational age at birth †§**	**Hazard ratio**	**Lower confidence limit**	**Upper confidence limit**	***P*‡**	
Historical vs prospective arm	1.04	0.91	1.19	0.58	Prospective
Maternal age	1.00	0.99	1.01	0.93	
Nulliparous vs parous	1.18	1.08	1.29	<0.001	Parous
Without vs with opioid use	1.10	0.88	1.37	0.41	With

GAB, gestational age at birth; NICU, neonatal intensive care unit; NICU LOS, NICU length of stay; NMI, neonatal morbidity and mortality index score; NNLOS, neonatal hospital length of stay.

*NNLOS and NICU LOS are reported for individuals in the 8.5% quantile of longest stays in each arm.

†GAB is reported for the earliest 8.5% quantile of births in each arm.

‡P- values report the significance of the individual covariates listed, and outcome comparisons between the prospective and historical arms are adjusted for these covariates.

§Cox proportional hazards regression analysis.

¶Ordinal logistic regression analysis.

Table S4. Exploratory analysis of neonatal morbidity and mortality index score (NMI) severity level probabilities and percent differences between arms for a range of covariate values in the intent-to-treat population.

				Predicted probabilities of NMI categories							Percent reduction in risk: Prospective versus historical arm	
Arm	Parous	Age	Opioid Use	NMI = 0	NMI = 1	NMI = 2	NMI = 3	NMI = 4	NMI ≥ 3	NMI ≥ 1	NMI ≥ 3	NMI ≥ 1
Historical	No	30	No	0.831	0.082	0.055	0.024	0.009	0.033	0.169		
Prospective	No	30	No	0.858	0.069	0.045	0.020	0.007	0.027	0.142	18.47%	16.29%
Historical	Yes	30	No	0.894	0.053	0.034	0.014	0.005	0.020	0.106		
Prospective	Yes	30	No	0.912	0.044	0.028	0.012	0.004	0.016	0.088	18.68%	17.32%
Historical	No	30	Yes	0.652	0.147	0.119	0.060	0.022	0.082	0.348		
Prospective	No	30	Yes	0.698	0.132	0.102	0.050	0.018	0.068	0.302	17.69%	13.24%
Historical	Yes	30	Yes	0.763	0.109	0.078	0.036	0.013	0.050	0.237		
Prospective	Yes	30	Yes	0.799	0.095	0.066	0.030	0.011	0.041	0.201	18.21%	15.16%
Historical	No	20	No	0.842	0.077	0.051	0.023	0.008	0.031	0.158		
Prospective	No	20	No	0.868	0.065	0.042	0.018	0.006	0.025	0.132	18.51%	16.47%
Historical	Yes	20	No	0.901	0.049	0.031	0.013	0.005	0.018	0.099		
Prospective	Yes	20	No	0.919	0.041	0.026	0.011	0.004	0.015	0.081	18.70%	17.44%
Historical	No	20	Yes	0.670	0.141	0.113	0.056	0.021	0.076	0.330		
Prospective	No	20	Yes	0.715	0.127	0.096	0.046	0.017	0.063	0.285	17.79%	13.56%
Historical	Yes	20	Yes	0.777	0.103	0.073	0.034	0.012	0.046	0.223		
Prospective	Yes	20	Yes	0.812	0.090	0.061	0.028	0.010	0.038	0.188	18.27%	15.40%
Historical	No	40	No	0.819	0.087	0.059	0.026	0.009	0.036	0.181		
Prospective	No	40	No	0.848	0.074	0.049	0.022	0.008	0.029	0.152	18.43%	16.10%
Historical	Yes	40	No	0.886	0.057	0.036	0.016	0.005	0.021	0.114		
Prospective	Yes	40	No	0.906	0.047	0.030	0.013	0.004	0.017	0.094	18.65%	17.19%
Historical	No	40	Yes	0.633	0.152	0.126	0.065	0.024	0.089	0.367		
Prospective	No	40	Yes	0.680	0.138	0.109	0.053	0.020	0.073	0.320	17.59%	12.91%
Historical	Yes	40	Yes	0.748	0.115	0.084	0.039	0.014	0.054	0.252		
Prospective	Yes	40	Yes	0.785	0.100	0.070	0.032	0.012	0.044	0.215	18.15%	14.91%
